# Ablation of Neurogenesis Attenuates Recovery of Motor Function after Focal Cerebral Ischemia in Middle-Aged Mice

**DOI:** 10.1371/journal.pone.0046326

**Published:** 2012-10-26

**Authors:** Fen Sun, Xiaomei Wang, XiaoOu Mao, Lin Xie, Kunlin Jin

**Affiliations:** 1 Department of Pharmacology & Neuroscience, Institute for Aging and Alzheimer's Disease Research, University of North Texas, Fort Worth, Texas, United States of America; 2 Buck Institute for Research on Aging, Novato, California, United States of America; University of South Florida, United States of America

## Abstract

Depletion of neurogenesis worsens functional outcome in young-adult mice after focal cerebral ischemia, but whether a similar effect occurs in older mice is unknown. Using middle-aged (12-month-old) transgenic (DCX-TK^(+)^) mice that express herpes simplex virus thymidine kinase (HSV-TK) under control of the doublecortin (DCX) promoter, we conditionally depleted DCX-positive cells in the subventricular zone (SVZ) and hippocampus by treatment with ganciclovir (GCV) for 14 days. Focal cerebral ischemia was induced by permanent occlusion of the middle cerebral artery (MCAO) or occlusion of the distal segment of middle cerebral artery (dMCAO) on day 14 of vehicle or GCV treatment and mice were killed 24 hr or 12 weeks later. Increased infarct volume or brain atrophy was found in GCV- compared to vehicle-treated middle-aged DCX-TK^(+)^ mice, both 24 hr after MCAO and 12 weeks after dMCAO. More severe motor deficits were also observed in GCV-treated, middle-aged DCX-TK^(+)^ transgenic mice at both time points. Our results indicate that ischemia-induced newborn neurons contribute to anatomical and functional outcome after experimental stroke in middle-aged mice.

## Introduction

Aging is associated with a striking increase in the incidence of stroke and neurodegenerative diseases, which are major causes of disability among those aged 70 years and older [1], [2]. Although stroke in humans usually afflicts the elderly [1], [2], most experimental studies of cerebral ischemia have employed young-adult animals, due to their greater availability, lower cost and fewer health problems [3], [4], [5]. Abnormalities in glycolytic flux, lactate production, oxidation and energy production are more pronounced with advancing age, indicating a reduced ability of the brain to adapt to stress [6]. Consistent with these observations, ischemic changes are more pronounced with advancing age in models of both global and focal cerebral ischemia [2], [6], [7], [8], [9]. Consequently, ischemia models that may be more relevant to human stroke have been developed using older rats and mice [7], [9], [10], [11], [12], [13], [14], which thus are helpful for evaluating new strategies for stroke therapies.

In the adult brain, neurogenesis occurs in the subventricular zone (SVZ) of the lateral ventricles, from where newly generated cells migrate into the olfactory bulb, and the subgranular zone (SGZ) of the dentate gyrus (DG), where new granule cells become integrated into the local neuronal network. Neural stem cells (NSCs) in these regions proliferate in response to focal ischemia [4] and global ischemia [5] in young-adult animals. The newborn cells can subsequently migrate into damaged brain regions [15], where they differentiate into cells with phenotypic properties of mature neurons [16], [17], [18]. Recent studies show that neurogenesis diminishes with aging [19], [20], [21], [22], and this limit the suitability of the aged brain as a target for cell replacement therapy. However, we and others have found that despite an age-related reduction in basal NSCs proliferation, NSCs in the SVZ of aged rats [8] and the penumbral region surrounding the ischemic core in human stroke brain [23] retain the capacity for proliferation and localization to ischemic lesions, although the response is less robust than in younger animals. Interestingly, the absolute number of stroke-generated new striatal neurons is similar in young-adult and aged rats [24]. Our previous study showed that depletion of doublecortin (DCX)-positive cells in 3-month-old ganciclovir (GCV)-treated transgenic mice that express herpes simplex virus thymidine kinase (HSV-TK) under control of the DCX promoter increased infarct size and exacerbated postischemic sensorimotor behavioral deficits [25]. Whether newly-generated neurons also contribute to functional outcome in older animal after experimental stroke remains unclear.

**Figure 1:**
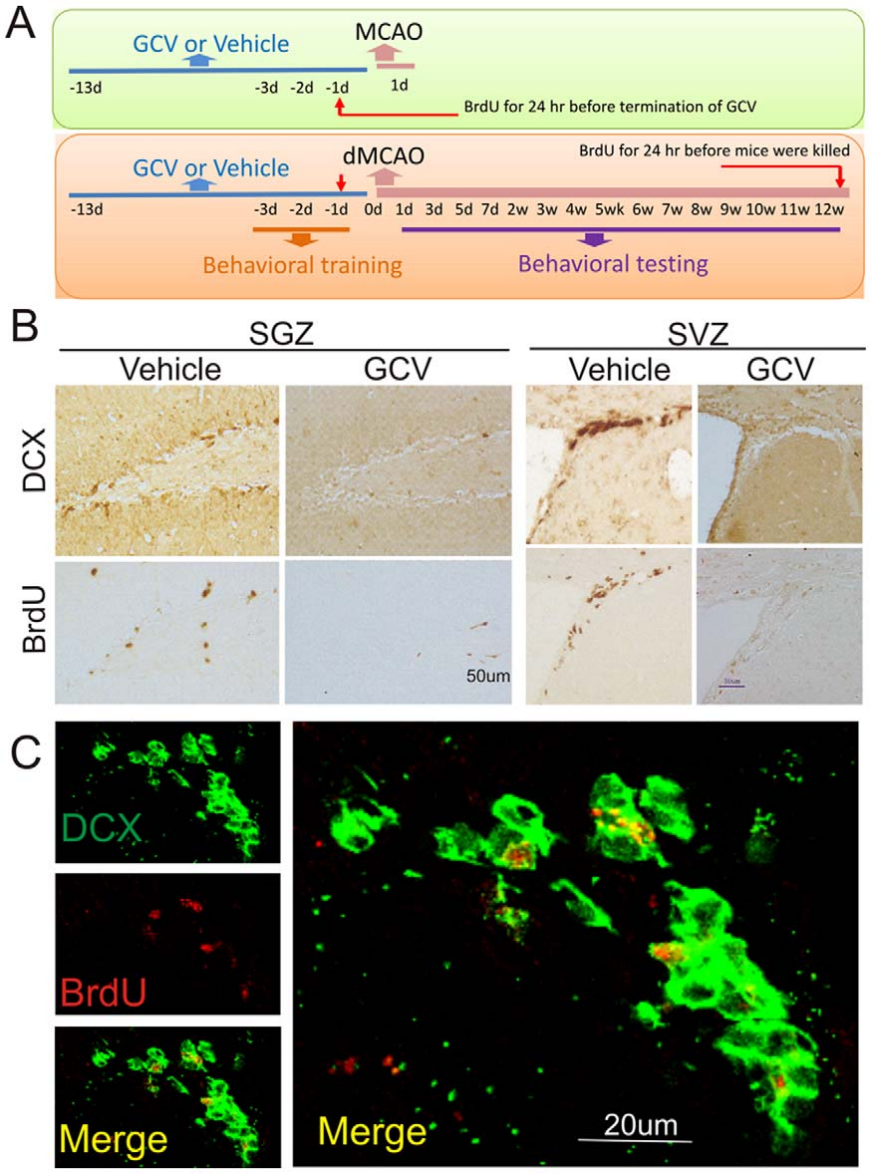
Conditional depletion of DCX- and BrdU-immunopositive cells in SVZ and dentate SGZ of middle-aged DCX-TK^(+)^ transgenic mice. (**A**) Middle-aged DCX-TK^(+)^ and DCX-TK^(−)^ mice were treated for 14 days with vehicle or GCV, received behavioral training, and then underwent MCAO or dMACO. Behavioral testing was conducted for 12 weeks after dMCAO, following which some mice were given BrdU on the last day after dMCAO. Mice were then euthanized for measurement of damaged volume and immunocytochemistry. In addition, BrdU was injected at 24 hr prior to termination of GCV administration to test whether neurogenesis was inhibited. (**B**) Representative images of DCX- and BrdU-immunopositive cells in dentate SGZ of middle-aged DCX-TK^(+)^ transgenic mice treated with vehicle or GCV (left panel). DCX- and BrdU-immunopositive cells in SVZ of middle-aged DCX-TK^(+)^ transgenic mice treated with vehicle or GCV (right panel). (**C**) Representative images of DCX (green)/BrdU-immunopositive cells (red) in SVZ of middle-aged DCX-TK^(+)^ transgenic mice treated with vehicle (left pane). High magnification view (right panel) shows that DCX (green) and BrdU (red) were colocalized in single cells.

In the current study, we conditionally depleted newly generated neurons in middle-aged mouse and assessed outcome after focal cerebral ischemia. We found that depletion of DCX-expressing cells in GCV-treated DCX-TK^(+)^ middle-aged mice resulted in both increased infarct volume (acute phase) or brain atrophy (chronic phase) and more severe neurologic deficits, compared with vehicle-treated DCX-TK^(+)^ middle-aged mice and either GCV- or vehicle-treated DCX-TK^(−)^ mice. Our results indicate that ischemia-induced new-born neurons also contribute functional recovery in middle-aged mice after experimental stroke.

**Figure 2:**
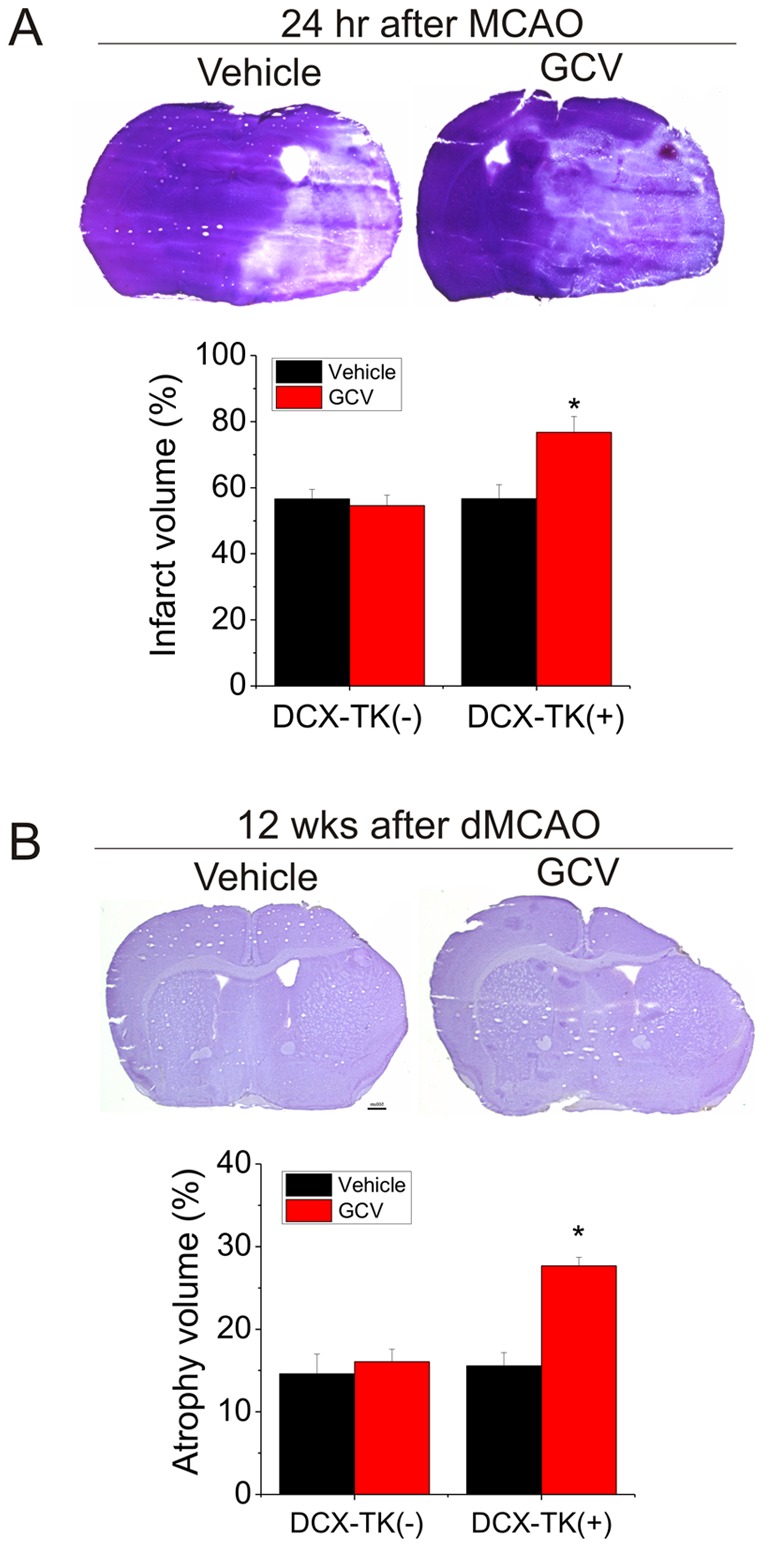
Infarct volume or volume loss in middle-aged DCX-TK^(−)^ and DCX-TK^(+)^ transgenic mice after dMCAO. (**A**) Middle-aged DCX-TK^(−)^ and DCX-TK^(+)^ transgenic mice were treated with vehicle or GCV for 14 days, underwent MCAO, and were killed 24 hr later. Top panel: representative images of infarct area in H&E-stained coronal brain sections. Bottom panel: infarct volumes, expressed as percentage hemispheric volume. *P<0.05. (**B**) Middle-aged DCX-TK^(−)^ and DCX-TK^(+)^ transgenic mice were treated with vehicle or GCV for 14 days, underwent dMCAO, and were killed 12 weeks later. Top panel: representative images of atrophy area in H&E-stained coronal brain sections. Bottom panel: volume loss, expressed as percentage hemispheric volume. *P<0.05.

## Materials and Methods

### Production of DCX-TK(+) transgenic mice

Transgenic CD1 mice (DCX-TK^(+)^) that express HSV-1 TK under control of the DCX promoter and DCX-TK^(−)^ mice were generated at Buck Institute for Research on Aging as described in our previous publication [25]. All procedures were approved by Institutional Animal Care and Use Committee and conducted according to the National Institutes of Health (NIH) Guide for the Care and Use of Laboratory Animals.

**Figure 3:**
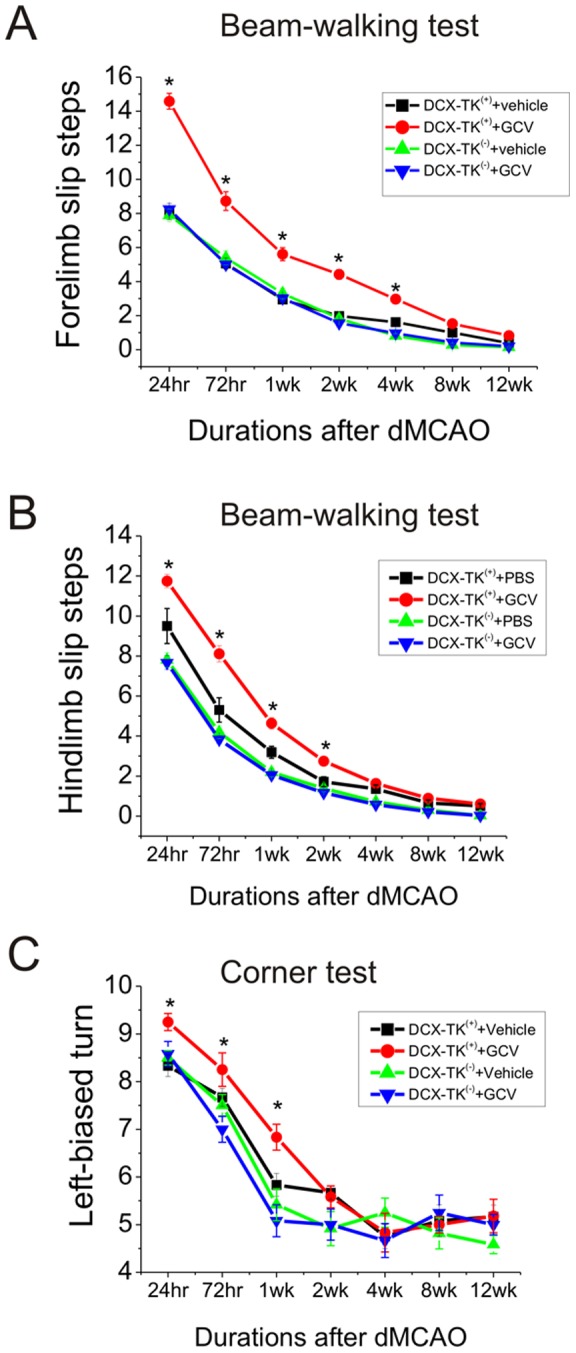
Beam-walking and corner testing in middle-aged DCX-TK^(−)^ and DCX-TK^(+)^ mice after dMCAO. Middle-aged DCX-TK^(−)^ and DCX-TK^(+)^ mice underwent dMCAO after treated with vehicle or GCV for 14 days, then behavioral tests were conducted at intervals over the next 12 wks (weeks). (**A**) Forelimb slip steps of Beam-walking test (higher scores correspond to more severe deficits). (**B**) Hindlimb slip steps of Beam-walking test (higher scores correspond to more severe deficits). (**C**) Scores of corner test (higher scores correspond to more severe deficits). *P<0.05.

**Figure 4:**
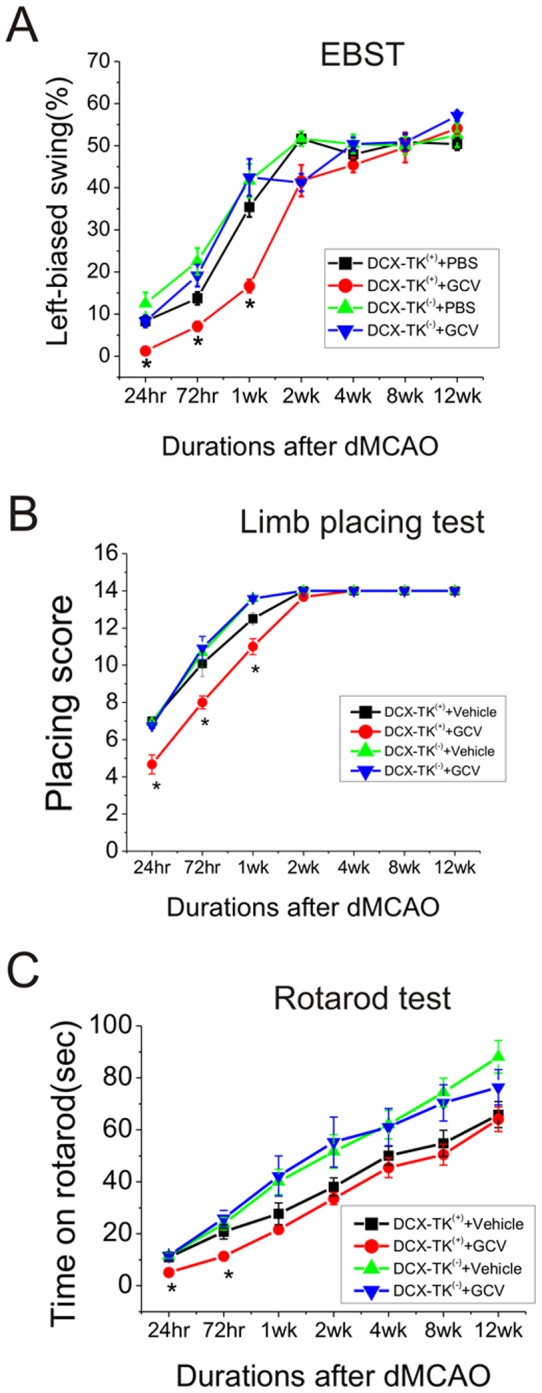
EBST, limb-placing and rotarod testing in middle-aged DCX-TK^(−)^ and DCX-TK^(+)^ mice after dMCAO. Middle-aged DCX-TK^(−)^ and DCX-TK^(+)^ mice underwent dMCAO after treated with vehicle or GCV for 14 days, then behavioral tests were conducted at intervals over the next 12 wks. (**A**) Scores of elevated body swing test (EBST; lower scores correspond to more severe deficits). (**B**) Scores of limb-placing test (lower scores represent more severe deficits). (**C**) Scores of rotarod test (lower scores represent more severe deficits). *P<0.05.

### GCV administration

Middle-aged (12-month-old) male DCX-TK^(+)^ or DCX-TK^(−)^ mice were anesthetized with 4% isoflurane in 70% N_2_O/30% O_2_, implanted with osmotic minipumps (Alzet, Cupertino, CA, USA), and infused continuously for 14 days with 0.25 μl/hr of either 20 mM ganciclovir (GCV) (Roche, Indianapolis, IN, USA) or vehicle (PBS). Focal ischemia was induced on day 14 of GCV administration.

**Figure 5:**
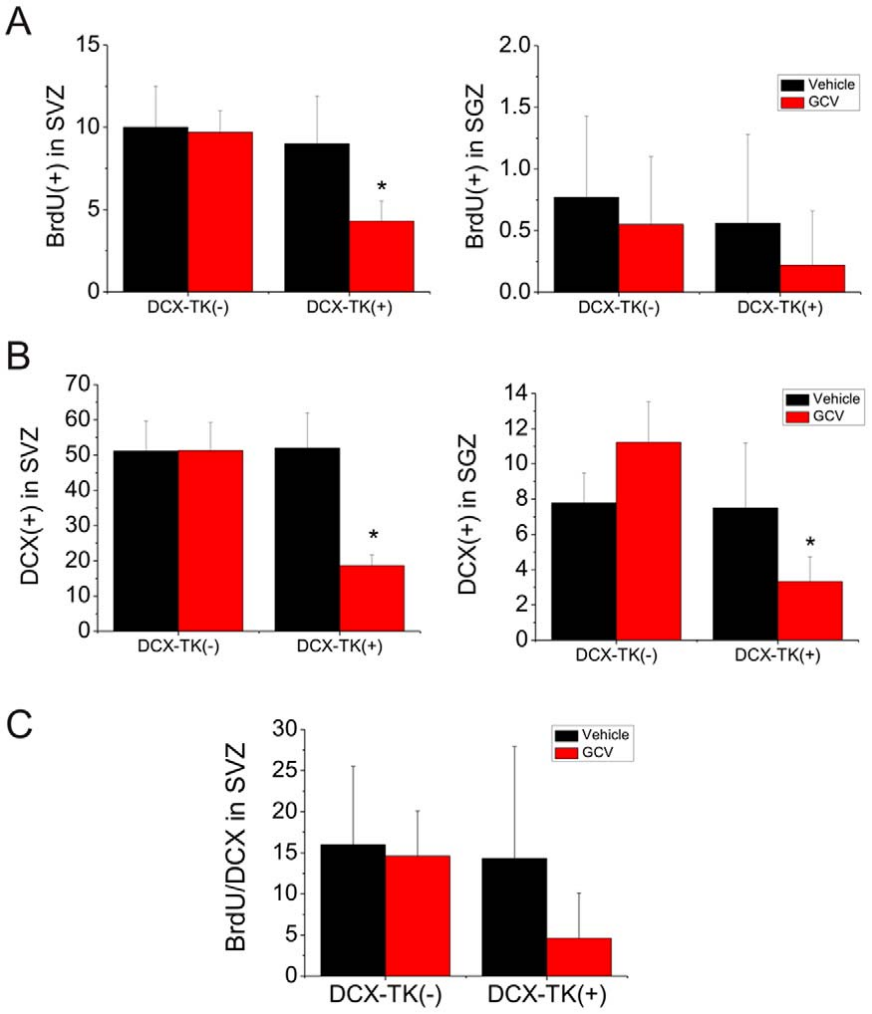
DCX- and BrdU-positive cells in the SVZ and SGZ 12 weeks after depletion of neurogenesis followed dMCAO. (**A**) Qualification of BrdU-immunoreactive cells in the SVZ (left panel) and SGZ (right panel) of vehicle- or GCV-treated middle-aged DCX-TK^(−)^ and DCX-TK^(+)^ mice at 12 weeks after dMCAO. *P<0.05. (**B**) Qualification of DCX-immunoreactive cells in the SVZ (left panel) and SGZ (right panel) of vehicle- or GCV-treated middle-aged DCX-TK^(−)^ and DCX-TK^(+)^ mice at 12 weeks after dMCAO. *P<0.05 (**C**) Qualification of DCX/BrdU-positive cells in the SVZ of vehicle- or GCV-treated DCX-TK^(+)^ and DCX-TK^(−)^ mice at 12 weeks after depletion of neurogenesis.

### BrdU administration

5-bromo-2-deoxyuridine (BrdU; Sigma-Aldrich, St. Louis, MO, USA; 50 mg/kg) was dissolved in saline and injected intraperitoneally twice daily at 24 hr prior to termination of GCV administration or on the last day after dMCAO and animals were killed 24 h later.

### Permanent focal cerebral ischemia

Middle-aged (12-month-old) male mice (DCX-TK^(+)^ and DCX-TK^(+)^) were anesthetized with 2.0% isoflurane in 70% N_2_O/30% O_2_ using a vaporizer. Permanent intraluminal suture occlusion of the middle cerebral artery (MCAO) and distal middle cerebral artery occlusion (dMCAO) were performed for short-term and long-term outcome assessment respectively. MCAO was induced by intraluminal occlusion with a nylon monofilament suture as described previously [25]. For dMCAO, after making a 1-cm skin incision between the left eye and ear, a burr hole was drilled through the temporal bone. The dura mater was removed and the middle cerebral artery (MCA) was occluded permanently using a bipolar electrocoagulation forceps. Interruption of cerebral blood flow was confirmed visually under a microscope and by laser-Doppler flowmetry (Moor Instruments, Wilmington, DE, UK). During the operation, rectal temperature was maintained at 37±0.5°C with a thermostat-controlled heating blanket (Harvard Apparatus, Holliston, MA, USA). After suturing the skin lesion, the mice were placed in a cage under an infrared heating lamp until they recovered from anesthesia. Sham-operated mice underwent identical surgery except that the MCA was not occluded. Brains were perfused with 0.9% saline and 4% paraformaldehyde in PBS (pH 7.4) and embedded in paraffin. Some brains were also freshly isolated, and 50-μm coronal sections were cut with a cryostat for histology analysis (hematoxylin and eosin stain; H&E stain).

### Immunohistochemistry

Immunohistochemistry was performed as described previously [25]. To detect BrdU-labeled cells, sections (6 mice/group) were incubated in cold methanol at −20°C for 10 min, washed in PBS for 3 min, and then treated with 2 M HCl at 37°C for 60 min, following rinsing in 0.1 M boric acid (pH 8.5) and PBS. Sections were then incubated in 1% H_2_O_2_ for 15 min and in blocking solution (2% goat serum, 0.3% Triton X-100, and 0.1% BSA in PBS) for 2 hr at room temperature, before being treated with mouse monoclonal anti-BrdU overnight at 4°C. Immunohistochemistry was conducted using a standard protocol with antigen retrieval (Vector Laboratories, Burlingame, CA, USA), according to the manufacturer's instructions. The primary antibodies used were mouse monoclonal anti-BrdU (1∶1000; Roche, South San Francisco, CA, USA) and affinity-purified goat anti-DCX (1∶200; Santa Cruz Biotechnology, Santa Cruz, CA, USA). The second antibodies were biotinylated donkey anti-goat or biotinylated horse anti-mouse antibody (all 1∶200; Santa Cruz Biotechnology). Sections were examined with a Nikon E800 epifluorescence microscope. Controls included omitting the primary or secondary antibody.

### Double-label immunohistochemistry

Double-label immunohistochemistry (5 mice/group) was performed as described elsewhere [25]. Primary antibodies were those described above; secondary antibodies were Alexa Fluor 488-conjugated goat anti-mouse (for BrdU) or Alexa Fluor 594- donkey anti-goat (for DCX) (1∶200–500; Molecular Probes, Eugene, OR, USA). Nuclei were counterstained with 4′,6-diamidino-2-phenylindole (DAPI) using the proLong Gold antifade reagent (Molecular Probes). Fluorescence signals were detected using an LSM 510 NLO Confocal Scanning System mounted on an Axiovert 200 inverted microscope (Carl Zeiss Ltc., Thornwood, NY, USA) equipped with a two-photon Chameleon laser (Coherent Inc., Santa Clara, CA, USA), and images were acquired using LSM 510 Imaging Software (Carl Zeiss). Selected images were viewed at high magnification. Controls included omitting either the primary or secondary antibody.

### Cell counting

BrdU- and DCX-positive cells in the SVZ and DG were counted by an observer blind to experimental condition using a Zeiss microscope in bright field mode and a 40 x objective. A two-photon confocal microscope was used to count double-labeled cells. In the SVZ, DCX- and BrdU-labeled cells were counted along the lateral walls of the lateral ventricles, corresponding to coronal coordinates interaural 8.7 to 10.2 mm, bregma −0.30 to bregma −1.2 mm, for a total of 6-µm coronal sections (n = 6; 100 µm apart) per mouse (6 mice per group for single immunostaining and 5 mice per group for double immunostaining). In the DG, DCX- and BrdU-labeled cells within two cell diameters from the inner edge of the granule cell layer (GCL) of the dente gyrus (interaural 4.48 to 5.86 mm, bregma −4.52 to bregma −3.14) were included in the analysis.

### Histology

DCX-TK^(+)^ or DCX-TK^(−)^ mice (n = 4) were anesthetized and decapitated 24 hr after MCAO or 12 weeks after dMCAO. Brains were removed and 50-µm coronal sections were prepared and stained with H&E. Infarct area (24 hr after MCAO) or brain atrophy area (12 weeks after dMCAO), left hemisphere area, and total cross-sectional brain area were measured by a blinded observer using the NIH Image program, and areas were multiplied by the distance between sections to obtain the respective volumes. Infarct volume or volume loss (brain atrophy) was calculated as a percentage of the volume of the contralateral hemisphere, as described previously [26].

### Beam-walking test

The beam-walking test was used to assess deficits in coordination and integration of motor movement, especially in the hindlimb [27]. The beam-walking test was performed as previously described with modification [27], [28]. In briefly, the mice (12 mice/group) were trained to traverse a wooden beam (8 mm wide, 1.2 m long and 45 cm high) into a darkened plastic box for 3 consecutive days before the induction of ischemia and by the end of the training period all mice had learned the task. Each test session consisted of four trials (two trials in each direction), in which latency to cross the beam and the number of forelimb and hindlimb foot faults was recorded. A fault was defined as any foot slip off the top surface of the beam or any limb use on the side of the beam. Four trials were averaged to give a mean foot fault score, and testing was performed 24 hr, 72 hr, and 1, 2, 4, 8 and 12 weeks after ischemia.

### Corner test

The corner test, used to test integrated sensorimotor function, was performed according to a previous protocol [29]. Briefly, the mouse (12 mice/group) was placed between two cardboards, each with a dimension of 30×20×1 cm^3^. The two boards were gradually moved to enclose the mouse from both sides and encourage the mouse to enter into a 30° corner with a small opening along the joint between the two boards. When the mouse entered deep into the corner, both the vibrissae on both sides were stimulated by the boards. The mouse then reared forward and upward, and then turned back to face the open end. Twenty trials were performed for each mouse and the percentage of left turns was calculated. Only turns involving full rearing along either board were recorded.

### Elevated body swing test (EBST)

The elevated body swing test was used to test asymmetric motor behavior. Mice (n = 12 per group) held by the base of the tail were raised ≈10 cm above the testing surface. The initial direction of body swing, constituting a turn of the upper body of >10° to either side, was recorded in three sets of 10 trials, performed over 5 min. The number of turns in each (left or right) direction was recorded, and the percentage of turns made to the side contralateral to the ischemic hemisphere (percent left-biased swing) was calculated. For each mouse, average scores were determined.

### Limb-placing test

Limb-placing, a test of lateralized post-ischemic sensorimotor dysfunction [30], was evaluated bilaterally from 24 hr to 12 weeks after focal ischemia. The test consisted of seven limb-placing tasks, which were scored by a blinded observer as follows: 0, no placing; 1, incomplete or delayed placing; 2, complete and immediate placing. Forelimb and hindlimb scores were averaged for each animal (12 mice/group).

### Rotarod test

Mice (12 mice/group) were trained on an accelerating (5 to 40 rpm) rotating rod (rotarod) for 3 days before focal ischemia; only those mice able to remain on the rod for 20 s at 40 rpm were subjected to focal ischemia [31]. Test sessions consisting of three trials at 40 rpm were carried out just before focal ischemia, and at 24 hr to 12 weeks after focal ischemia, by an investigator who was blinded to the experimental groups. The final score was expressed as the mean time that a mouse was able to remain on the rod over three trials.

### Statistical analyses

Quantitative data were expressed as mean ± SEM from the indicated number of experiments. Behavioral data were analyzed by two-way analysis of variance (ANOVA) with repeated measures, followed by *post hoc* multiple comparison tests (Fisher PLSD or Student's paired *t* test with the Bonferroni correction). Brain atrophy data were analyzed by one-way ANOVA followed by Fisher PLSD *post hoc* tests. *P* values <0.05 were considered significant.

## Results

Middle-aged DCX-TK^(+)^ and DCX-TK^(−)^ mice were treated with GCV or vehicle for 14 days and received behavioral training, and then underwent MCAO or dMACO. Behavioral testing was conducted for 12 weeks after dMCAO (**Figure 1A**). Mice were then euthanized for measurement of damaged volume and immunocytochemistry. As shown in **Figure 1B**, DCX-positive cells were found in the SVZ and dentate SGZ of middle-aged DCX-TK^(+)^ transgenic mice treated with vehicle. After treatment with GCV for 14 days, DCX- and BrdU-positive cells in SVZ and SGZ were barely detectable middle-aged DCX-TK^(+)^ transgenic, compared to DCX-TK^(−)^ mice. Double immunostaining shows that most BrdU-positive cells expressed DCX (**Figure 1C**). Thus, GCV treatment depleted NSCs from the middle-aged mouse brains.

To determine the role of neurogenesis in outcome from focal ischemia in middle-aged mice, we first induced focal ischemia by permanent intraluminal suture occlusion of MCA after administration of GCV or vehicle for 14 days, and mice were killed 24 hr later. As shown in **Figure 2A**, infarct volume was significantly increased in DCX-TK^(+)^ transgenic mice treated with GCV, compared to vehicle-treated DCX-TK^(+)^ mice and either vehicle- or GCV- treated middle-aged DCX-TK^(−)^ mice, suggesting that NSCs in middle-aged brain influence acute histological outcome after MCAO.

To determine if depletion of DCX-positive cells could also affect long-term histological outcome, we induced focal cerebral ischemia by permanent dMCAO in middle-aged mice treated with GCV or vehicle for 14 days. dMCAO was used in these experiments because it produces stroke mortality is generally lower in older rats [32], and a smaller infarct permits longer survival. Our post-stroke mortality rate in the dMCAO model was very low–about 10%. As shown in **Figure 2B**, volume loss at 12 weeks in GCV-treated middle-aged DCX-TK^(+)^ mice was significantly higher than that in vehicle-treated DCX-TK^(+)^ and vehicle- or GCV-treated DCX-TK^(−)^ mice.

Next we measured neurological deficits in middle-aged DCX-TK^(+)^ mice. We found that deficits in beam-walking (**Figure 3A and B**), corner tests (**Figure 3C**), elevated body swing (**Figure 4A**), limb-placing (**Figure 4B**), and rotarod (**Figure 4C**) were worse in the GCV-treated middle-aged DCX-TK^(+)^ transgenic mice tested up to 4 weeks after dMCAO, compared to vehicle-treated DCX-TK^(+)^ and vehicle- or GCV-treated DCX-TK^(−)^ mice, suggesting involvement of newly generated cells in functional outcome after focal ischemia.

Finally, we asked whether NSCs in the SVZ and SGZ of dentate gyrus were able to restore newborn neurons in middle-aged mice after nearly complete ablation of DXC-positive cells following focal ischemia. As shown in **Figure 5A and B**, the DCX- and BrdU-positive cells in the SVZ and SGZ of hippocampus, which were depleted after GCV treatment, were partially restored up to ∼20% in 12 weeks after depletion. Double-label immunohistochemistry confirmed that about 16% DCX-positive cells in the SVZ of middle-aged DCX-TK^(−)^ mice treated with GCV or vehicle incorporated into BrdU, but only less than 5% DCX-positive cells in the SGZ of dentate gyrus incorporated into BrdU (**Figure 5C**) in middle-aged DCX-TK^(+)^mice treated with GCV. Collectively, these results indicate that neurogenesis in middle-aged brain can be slowly restored to normal baseline at certain recovery period even after severely depletion.

## Discussion

In the current study, we found that the NSC depletion causes larger infarct volumes or volume loss and increases neurological deficits in middle-aged mice after focal cerebral ischemia. This suggests that ischemia-induced neurogenesis exerts a beneficial effect on short-term and long-term outcome not only in young-adult (25), but also in middle-aged mice.

Brain damage, such as stroke, triggers reparative mechanisms, among which neurogenesis may be one. Experimental (4) and clinical (23) stroke are both associated with increased neurogenesis, and the time course over which occurs coincides with the most active period of functional recovery. Although this does not necessarily imply a causal relationship between neurogenesis and recovery, inhibition of neurogenesis by antimitotic drugs is associated with worsened outcome after cerebral ischemia [33]. In a previous study (25), we generated conditional DCX-TK^(+)^ transgenic mice, in which neurogenesis could be selectively ablated after administration of GCV. Using this approach, we found that depletion of newborn cells in young-adult mice results in worsened functional outcome after experiment stroke. As stroke predominantly occurs in more aged populations [34], stroke models using older animals may be clinically more relevant than stroke models in young-adults.

Although basal neurogenesis in the SVZ and SGZ of dentate gyrus declines with age, we found previously that SVZ neurogenesis is still increased after focal ischemia, albeit to a lesser extent, in older rats, who therefore appear to retain the capacity for stroke-induced neurogenesis [8]. Consistent with this, another study confirmed that the number of stroke-generated new striatal neurons was similar in young-adult and aged rats [24]. Our findings in the present study suggest that newly generated NSCs contribute significantly to functional recovery after experimental stroke in middle-aged rodents. This raises the possibility that NSCs might be beneficial in the clinical treatment of stroke, even though–because clinical recovery is often incomplete–the innate capacity for brain repair after stroke appears to be limited. We found that infarction volume were significantly larger at 24 hours in DCX-TK(+) mice, compared with DCX-TK(−) mice. Although the exact mechanisms underlying improved functional and histological outcomes are incompletely unclear, neuroprotection may play an important role, as NSCs are known to constitutively produce neurotrophic factors such as nerve growth factor (NGF) and glial cell line-derived neurotrophic factor (GDNF) [35]. Notably, significant recovery occus in both DCX-TK(+) and DCX-TK(−) mice several weeks after dMCAO, which may be due to “self-repair” after brain injury, including restoration of neurogenesis.

Neurogenesis in the aging brain can be manipulated by pharmaceutical tools, such as growth factors. A recent study showed that granulocyte-colony stimulating factor (G-CSF), a cytokine, exerts a robust and sustained beneficial effect on survival, motor function, and working memory after focal ischemia, and stimulates the proliferation of NSCs cells in the ipsilateral SVZ [36]. Other work indicates that the SVZ continues to produce new neuroblasts, which migrate into the striatum and then adopt features of a mature neuronal phenotype, for at least 4 months [37], and even up to 1 year [38], after 2 hours of focal cerebral ischemia in rats. Thus, despite the low basal rate of NSCs proliferation in the SVZ, NSCs in the aging brain could remain a vehicle for self-repair after stroke.
